# Dr. G. V. Black’s Conclusions Reviewed

**Published:** 1897-12

**Authors:** S. B. Palmer

**Affiliations:** Syracuse, N. Y.


### DR. G. V. BLACK'S CONCLUSIONS REVIEWED.[1]

BY DR. S. B. PALMER, SYRACUSE, N. Y.

My friend, Dr. Lord, recently made a request in your behalf, inviting me to give my conclusions upon the subjects which have been so ably discussed by Dr. Black, which I am informed are to be further discussed at your next meeting. I regret to offer my own opinions in opposition to one of authority and one so widely known and appreciated as a scientific investigator as Dr. Black. I have enjoyed Dr. Black's experiments, as given before the State Society, and more especially in his room at Albany, upon the subject of alloys and amalgams. Tests for shrinkage, edge strength, flow, etc., were made of numerous alloys, manipulated by different persons so accurately, and recorded by instruments so perfectly that all beholders were convinced and the results became knowledge to be utilized.

Dr. Black's investigations upon the physical characteristics of the teeth from a *physical* stand-point may be equally scientific, and I presume that others would record the same figures in repeating the experiments. Thus I will consume no time in an attempt to refute his tables. In a previous article the writer stated that his investigations were based upon the *organic* plane, consequently the teeth of children, or those which are not fully matured, are undergoing changes, consequently do not belong to the mineral kingdom to be classed with inert matter.

Time forbids taking up all the points mentioned in Dr. Black's conclusions. Perhaps the following are most derogatory to dental progress: "There is no basis for the selection and adaptation of filling-materials to soft teeth, hard teeth, frail teeth (in structure), or poorly calcified teeth. With our present knowledge, the only basis for selection and adaptation of filling-materials to classes of cases is the individual operator's judgment as to which he can so manipulate as to make the most perfect fillings, considering the circumstances, his own skill, and the durability of materials."

The above seems to indicate that operative dentistry has no scientific rules governing the proper adaptation of filling-materials to conditions of teeth. All is left to the operator's judgment. This may do for one who has had years of clinical experience, but to the young graduate from college, where such instructions have

[1] Read before The New York Institute of Stomatology, October 5, 1897.

been received, it amounts to license to practise in accord with the highest ambition of a promising student, and to fill all teeth with gold, regardless of its adaptability, which would violate a natural law, and the penalty would be the loss of teeth and loss of patient, as well as reputation.

Before receiving your request, I had thought this subject passed. It seems that "in the fulness of time" an object lesson has been given by which I may be understood, and I feel grateful for the opportunity to lay the matter before you. We will now take our stand upon the animal plane where man "lives, moves, and has his being," which is quite above the plane upon which the conclusions under discussion were based. The order is this: First, elements; second, minerals; third, vegetable; fourth, animal.

"There is but one science of chemistry. Its divisions into *inorganic* and *organic* is simply one of convenience."

There is also one fundamental law known as chemism, or chemical affinity, which has passed from the elementary, where its prominent manifestations are union of elements up to the mineral plane, where it has a broader field for action, and more material to act upon, and we see as a result chemical compounds all composed and decomposed; in short, electrolysis is established, electric currents are generated, laboratory work is performed, and all that pertains to investigation upon inert matter is upon the physical plane.

Following the same law with its added possibilities up to the vegetable, we find more material for evolution. The breadth of life has touched chemical compounds and vegetation springs up from lichen to forest-trees. Follow the fundamental law. The physical current of the second plane, which was directed by the experiments in electrolyses, has been *organized* and is presided over by vital force or direction. The physical battery, with its positive and negative plates, is not needed. By the action of the light upon the foliage and the rootlets in contact with moist soil, through the medium of the sap, a to-and-fro current is established, minerals are decomposed, and plant-food is furnished for the growth of vegetation. With this principle most readers are familiar, and very little use is made of the lessons given.

And this is the end of helps, so far as organic chemistry relates to the teeth. The law is lost sight of, and the great mystery of animal *life* appears in physiology, which is beyond comprehension, and thus experiments are conducted upon the physical, where theories can be demonstrated with mathematical accuracy, and scientifically as well, as the tables of experiments will show. The point I make is this: So far as the difference exists relating to the conditions of the teeth which we present, science, from the physical plane, is *misapplied.* Let us now follow the law with its aggregations to the animal, and see what material, what forces, and what director of forces we have to work with. Please pardon a seeming digression just here. I wish it understood that I do not attempt to meet the requirements demanded in an editorial in the January issue of *The Dental Practitioner*, 1896. The quotation shows one of the obstacles thrown in the way of this original writing, as well as to demonstrate that the investigations which we are called upon to refute are of the "earth earthy."

"Demonstrated facts cannot be successfully refuted by mere generalizations. They must be met by counter-facts as incontestably demonstrated by equally conclusive experiments. Physical proofs cannot be overthrown by metaphysical reasoning, and the world is waiting for some one to present a series of established tables that shall confute those of Dr. Black."

The above shows that the functions of the pulp in teeth have no influence upon the structure after twelve years of age. I quote the following:

"There is no basis for the supposition that some teeth are too soft or too poorly calcified to bear filling with gold or other metal in use for that purpose, since all are found to be abundantly strong."

"With our present knowledge the only basis for selection and adaptation of filling-materials is the operator's judgment as to which he can most perfectly manipulate."

There are facts recorded in the tables emanating from physical investigations so carefully prepared and systematically arranged that they are under the seal of science and challenge refutation. On the other hand, knowledge gained by clinical experience and correct observation becomes science as pure and true as that gained from experiments. Thus, since we cannot prove our position by presenting a series of established tables that shall refute those above quoted, we rest our case upon knowledge gained from clinical experience and systematic investigations upon the organic plane, and that it too bears the seal of science. This implies *knowledge*, and what do we know?

We know that teeth undergo changes from the time of their first calcareous deposit until the pulp, from natural limitation or from caries or accident, fails to perform its functions.

We know that teeth at twelve years of age have not reached the limit of pulp-nourishment.

We know that young teeth are softer than the same would be at maturity. Also that some are free from caries and others are badly decayed at the age of twelve or fourteen years.

We know that it is under a natural law that the latter decay when filled with gold without some insulating lining, for the following reasons: The fact that decay has attacked several teeth is evidence that the structure is faulty when the excavator fails to give the sound of cutting bone, the cuttings roll up as shavings, and we call the dentine soft. We know that the dentine is a conductor when in that condition. We realize that nature will not deposit lime salts against metal, and the approach to it is in proportion to the conductivity of the dentine. The line under fillings in normal dentine is hardly perceptible, and time produces no change detrimental to such fillings. On the other extreme, thermal changes prevent deposits or pulp-nourishment, and a thin lamina becomes devitalized, which is manifest in a year or more by a gray shade through the enamel; whereas, if gutta-percha or some non-irritating cement filling had been used the enamel would have remained clear.

I desire to fairly represent both phases or conditions of teeth, as we find them in general practice, notwithstanding the following quotation: "Caries of the teeth is not dependent upon any condition of the tissue of the teeth, but on conditions of their environment." We have endeavored to show that in young teeth pulps do have an influence upon sensitive dentine beneath metal fillings, and I will draw a line between dentine which is sensitive and that which is not, and pass the latter over to the physical plane where it properly belongs with inert matter. With experience in practice and from personal knowledge of metals in my own mouth, as given in previous papers, such a line must be drawn. The motive given for this laborious task seems to be for the abandonment of ideas and rules of practice which have been considered favorably in dental progress. Thus: "The greatest good that I could expect to come from this laborious investigation (except as it may affect the future study of the caries of the teeth) would be the general abandonment of the idea that the teeth of any patient are so lacking in density as to interfere with reasonable filling operations with any of the materials now in use."

I leave this last quotation without further comment, and close with a presentation of my views as to how the metals in the mouth affect sensitive dentine, pulps, and gums at the gingival border. For twenty years the labor has been almost in vain, so far as having the principles understood. The practical introduction of cataphoresis has enlightened operators upon electric currents to a degree so that my contribution can be understood. The object to be obtained in cataphoresis is to carry drugs into tissue, dentine, pulps, etc., which offer more or less resistance. An electric current passing through liquids, or moist substances, carries with it from positive to negative water and the drugs which it holds in solution, —cocaine with other drugs, placed in a cavity to which is applied an electrode; this electrode is charged with a current of electricity from batteries or modified currents taken from a central station. The application and likeness this system has to metals worn in the mouth as fillings, clasps, bridges, and crowns, is this: The metals mentioned are electrodes *simply*, not connected by an outside supply, and would be lacking of a current but for the favorable conditions present in the oral cavity to generate galvanic currents directly in contact with the electrode. Very little has been written or said about the electro-chemical action produced in mastication of food. Nature seems to have introduced this principle for pleasure in eating. Frequently in a single meal are found numerous positive and negative elements which, if properly arranged in a physical battery cell, would produce measurable currents. Added to the physical apparatus, the *oral cavity* contains elements which have been raised from minerals to food, preserving the same laws of government with the elements organized. The cell is lined with vital membrane. The saliva is an unstable fluid readily changed from positive to negative. The carbon appears in an organized form in coffee, toast, and broiled meat, acids in fruits, and compounds of acids and alkalies, with hot and cold drinks, are masticated and mixed, causing the electro-chemical action mentioned. Every electrician understands that a negative metal plate like gold when emersed in such a mass becomes charged with electricity; that is, its potential is above the other pole which consists of the best conductor nearest to the electrode of highest potential. The application is this: When a gold filling is inserted into normal dentine the structure is practically a non-conductor to a current of such low tension, and even in dentine of less density no trouble may arise. We are using a figure of conveying medicaments through resisting mediums. This is by a physical current such as is used in ordinary electrical experiments; at the same time it illustrates a principle in organic currents that has been denied by opponents. This principle is known as osmosis. This is well defined by Dr. Jack: "If we arrange a vessel containing a saline mixture on one side, and water on the other, with a permeable division between them, and apply the anode on the side of the salt and the cathode on the side of the water, the osmotic process is prevented. If, on the other hand, the anode is placed on the side of the water and the cathode in the salt, the osmotic action is greatly accelerated, and what otherwise would require hours to naturally effect is done immediately. This shows that electricity has the power to overcome or accelerate the natural law of osmosis." With these facts before us, and another still nearer the point, we wish to have it understood that we make the application. It is known that medicaments are carried with the current into lining tissue in the mouth. We know that the current seeks the best conductor or takes routes of least resistance, as a leak in the rubber and discharge into the gums gives evidence. In a recent article it was stated that twenty-five per cent. of gold-cap crowns and anchors of bridges produced an unhealthy condition where the metal came in contact with the gums. It was stated that it was not from impingement, as many suppose, but imperfection in the fit of the band, allowing accumulations, fermentation, etc. Even this does not afford a satisfactory answer. Knowledge which has been burned in, remains. Some years since I had a gold crown inserted over a molar which had become worn by a clasp after forty years support to a plate. The cap was straight or with parallel sides and a good fit. The gold extended slightly under the margin of the gums. No manifestation of impingement or irritation of the gums. The clasp was much like the crown except being split on one side and the cap or cover had a portion left open to give circulation so as not to confine food. In articulation with this below was a bridge of four crowns, coronal surfaces gold, which was a convincing proof of the theory advocated years before. I could fully realize that heat is convertible into electricity, for hot coffee more than any other hot drinks created a pain, not in the gum tissue, but in the dentine, to a degree that was exceedingly uncomfortable. Relief was obtained by the old standard remedy, *silver nitrate.*

Without the combinations and conditions to produce such results, and some knowledge of oral electricity, marked cases like the one described would have been passed by as having no foundation except in the imagination. The cause of the immediate sensitiveness was that the gold became charged by heat and the decomposition of saliva, which was as readily discharged into the gum tissue in contact with the dentine. Even this current differs but little from that generated out of the mouth, and the evil results are on the line of a leak in the insulator, except in the wearing of the apparatus the current is applied wherever food is masticated. Instead of the generator being located outside and the current conducted through insulated wires, the electrode is placed in the cavity as a filling or spud for bridge support, or around the necks of teeth by gold crowns. These electrodes are charged directly by the chemical mass during mastication. Without experience in this line the above results might not have been known. With the experience of the writer it becomes scientific knowledge that no physical tabulated experiments can refute.

We have mentioned the aid which cataphoresis may afford us in understanding electric currents in the mouth. In all chemical changes, such as masticating food in connection with saliva, electricity is generated. It is of the nature of the elements from which it is produced; that is, food which has been organized, or, we may say, is animal electricity, some portion of which may be taken up by the tissue, but most goes into the stomach with the food, where, during the process of digestion, it is absorbed or goes into the circulation. It is well known that when a negative plate like platinum or gold is immersed in a compound like food with saliva the plate becomes charged with electricity of the physical kind. This is known by those who wear metal plates. A taste is known that would not be perceived but for the metal, and that metal one that would not be acted upon by any of the chemicals in the mass. This process is simply changing a current which nature produced from organized elements, with no other taste than the agreeable sensation experienced in the mingling of opposite elements—acid and alkalies, in soda-water, sweet and sour in lemonade, the carbon in coffee, broiled meats, toasts, etc.—with saliva and other food. Any combination in the mouth by which the vital current is changed to the physical is disagreeable and unnatural. Approximate fillings of gold and amalgam is sufficient proof of this statement.

Let us now look at nature's process of development of the teeth. They are covered by the gums until enamelled. Eruption takes place; the enamel serves as an environment during the growth and calcification of dentine and the roots. The skin covers the body in like manner as the gums cover the enamel, or as the bark environs the trunk of a tree, the growth is from within. Abrasion or removal of the skin is attended with inflammation. By injury or puncture of the enamel dentine becomes sensitive. Filling is like an adhesive plaster on the abraded skin: it excludes external exciting agents and nature heals the wound. If it were possible to produce an elastic gold plaster for the skin, it would not answer without a non-conducting lining. The comparisons are parallel. The healing in the one case may be in a few weeks, in the other as many years according to development in the teeth.

Thus, a gold filling, large or small, which passes through enamel into dentine is an electrode charged, not by an outside system, but by the electricity in the mouth which gives it potential. It discharges according to the conductivity of the dentine. In normal dentine no discharge; in young teeth which are conductors, nature's process meets with an opposite current which in effect has been described above, showing that electricity has the power to overcome or accelerate the natural law of osmosis.

In comparing the effects of electric currents, the late Dr. Turner remarks, "Feeble agencies, operative for a long period, are often just as effective in affecting great changes as powerful agents at work during a short period."

There is no drug in connection with the gold filling, but an opposing counter-current, which overcomes natural law of osmosis, and in the cases and conditions of teeth favorable to such action the teeth receive no nourishment, and in effect animation is suspended.

Therefore, I believe in a scientific adaptation of filling-materials to conditions of the teeth.

---

